# Drooping Shoulders

**Published:** 1885-12

**Authors:** Dio Lewis


### DROOPING SHOULDERS.

This is a serious evil. It comprises both appearance and vitality. A stooping figure is not only a familiar expression of weakness or old age, but it is, when caused by careless habits, a direct cause of contracted chest and defective breathing. Unless you rid yourself of this crook while at school, you will probably go bent to your grave. There is one good way to cure it. Shoulder-braces will not help. One needs, not an artificial substitute, but some means to develop the muscles whose duty it is to hold the head and shoulders erect. I know of but one bull's eye shot. It is to carry a weight on the head. A sheepskin or other strong bag filled with twenty to eighty pounds of sand is a good weight. When engaged in your morning studies, either before or after breakfast, put this bag of sand on your head, hold your head erect, draw your chin close to your neck and walk slowly about the room, coming back, if you please, every minute or two to your book, or carrying the book as you walk. The muscles whose duty it is to hold the head and shoulders erect are hit, not with scattering shot, but with a rifle ball. The bones of the spine and the intervertebral substance will soon accommodate themselves to the new attitude. One year of daily practice with the bag, half an hour morning and evening, will give you a noble carriage, without interfering a moment with your studies.

It would be very difficult to put into a paragraph more important instruction than this. Your respiration, voice and strength of spine, to say nothing of your appearance, will find a new departure in this cure of drooping shoulders.—*Dio Lewis.*

